# Discovering antecedents of antisocial behavior in the classroom: the influence of social exclusion on antisocial risk-taking

**DOI:** 10.3389/fpsyg.2025.1625978

**Published:** 2025-08-14

**Authors:** Corinna Lorenz, Philipp Nicolay, Corinna Hank, Christian Huber, Nicola K. Ferdinand

**Affiliations:** ^1^Department of Psychology, University of Wuppertal, Wuppertal, Germany; ^2^School of Education, University of Wuppertal, Wuppertal, Germany

**Keywords:** antisocial risk-taking, social exclusion, classroom sociometry, social acceptance, moral disengagement

## Abstract

**Introduction/Background:**

This study investigates how social exclusion experiences influence antisocial risk-taking behaviors in adolescents by examining the interplay between classroom social acceptance and experimentally induced social exclusion.

**Methods:**

Using a sequential experimental design with students in years 7–9 of the German school system (ages 12–16), we first assessed participants’ classroom social acceptance within their classrooms through sociometric measures, then randomly assigned them to experience either experimentally induced social inclusion (*n* = 65) or exclusion (*n* = 64) using the Cyberball paradigm, and finally measured their antisocial risk-taking using an adapted Columbia Card Task as well as moral disengagement.

**Results:**

Results revealed a complex relationship whereby social exclusion effects were moderated by pre-existing classroom social acceptance status. Well-integrated adolescents responded to exclusion by reducing antisocial risk-taking when potential harm to others was high, while poorly integrated adolescents demonstrated the opposite pattern, increasing risky choices that could harm others. Our exploratory analysis further indicated that moral disengagement was positively associated with antisocial risk-taking and negatively correlated with classroom social acceptance, particularly among excluded adolescents.

**Discussion/Conclusions:**

These findings suggest that responses to social exclusion are not uniform but depend critically on adolescents’ established social status, contributing to our understanding of the cognitive and social factors that shape decision-making in antisocial contexts during this developmental period.

## 1 Introduction

Social exclusion, which fosters feelings of rejection and neglect, plays a critical role in shaping antisocial behaviors among students. Research suggests that antisocial behavior in students, including bullying, is often linked to experiences of victimization or neglect in familial and peer contexts (Leary et al., 2003; Pepler et al., 2008). We conceptualize antisocial behavior as actions that violate social norms and potentially cause harm to others, with bullying representing a particularly relevant and consequential manifestation of this behavior in educational settings. Bullying constitutes a pattern characterized by intentional harm-doing with a power imbalance and repetition over time (Olweus, 1993), often involving calculated actions to enhance one’s status or acquire resources at others’ expense (Peeters et al., 2010). Social exclusion, meanwhile, represents a form of peer rejection where individuals are deliberately left out of social interactions.

Our study focuses on how experiences of being excluded influence subsequent antisocial risk-taking tendencies. By operationalizing antisocial behavior through a risk-taking paradigm that captures decisions benefiting oneself while potentially harming others, we examine the cognitive processes underlying bullying tendencies. Social exclusion experiences may create a heightened sensitivity to potential future rejection, which influences how individuals interpret and respond to social situations (MacDonald and Leary, 2005; Newman, 2014; Smart Richman and Leary, 2009). Competing hypotheses explain reactions to social exclusion. For instance, the social reconnection theory posits that individuals may engage in cooperative behaviors to reintegrate into social groups (Maner et al., 2007), while the frustration-aggression hypothesis suggests that some individuals react with frustration and aggression (Stenseng et al., 2014).

The divergence in these responses highlights the complex nature of social exclusion as a social stressor that can trigger either adaptive efforts to rebuild social bonds or maladaptive antisocial reactions depending on various individual and contextual factors (Riva and Eck, 2016). This ambiguity is particularly relevant given the significant implications for educational settings where peer rejection and victimization are common experiences in everyday classroom situations, and the fact that extreme violence like school shootings have been associated with this repeated form of social rejection (Leary et al., 2003). Despite substantial research on these divergent reactions to social exclusion, it remains unclear what specific factors determine whether an individual will respond with antisocial behavior. Understanding the antecedents of antisocial tendencies is crucial for identifying which adolescents may be at risk for developing problematic behavioral patterns, especially since early antisocial tendencies can solidify into persistent behavioral issues across development (Calkins and Keane, 2009; Frick and Viding, 2009). Previous research has largely relied on correlational evidence, limiting our understanding of how exclusion experiences relate to subsequent antisocial behavior. In this study, we address this research gap by examining how both chronic and acute forms of social exclusion relate to antisocial behavior in adolescents, by specifically focusing on the interplay between established classroom social acceptance and behavioral responses to experimentally manipulated exclusion experiences. This approach allows us to disentangle the effects of long-term social status from immediate exclusion experiences, providing insight into the specific conditions under which acute exclusion experiences may be associated with antisocial tendencies.

Adolescence represents a critical period where neural systems undergo reorganization and social status becomes particularly important, making exclusion experiences especially impactful (Blakemore, 2018; Tomova et al., 2021). Experiences of social exclusion during adolescence can have profound effects on behavior, potentially triggering antisocial tendencies that manifest in classroom settings, according to a kind of “outcast-lash-out effect” (Reijntjes et al., 2010; Warburton et al., 2006). The social pain associated with exclusion can be particularly intense during this developmental stage due to adolescents’ heightened concern with social evaluation and peer approval (Blakemore and Mills, 2014; Somerville, 2013; Tomova et al., 2021). Research has shown that exclusion activates neural regions associated with physical pain, highlighting its significant psychobiological impact (MacDonald and Leary, 2005). This neurobiological response to exclusion may be amplified in adolescents who are already marginalized within their peer groups, creating a cumulative effect of social rejection experiences (Newman, 2014). Both immediate and long-term consequences of exclusion contribute to ongoing cycles of bullying and victimization, potentially creating self-reinforcing patterns where exclusion leads to antisocial behavior, which in turn leads to further rejection by peers (Arseneault et al., 2010; Averdijk et al., 2016; Leary et al., 2003; Reijntjes et al., 2010; Stenseng et al., 2014, 2015).

Laboratory studies on immediate consequences provide evidence for the dichotomy between anti- and pro-social tendencies in responses to exclusion: some individuals show reduced self-regulation with increased aggression (Baumeister et al., 2005; Twenge et al., 2001, 2007), while others display more cooperative, socially conforming behavior (Maner et al., 2007). These divergent reactions suggest that individual differences in temperament, cognitive processing, and social experiences may moderate how acute exclusion affects behavior (Riva and Eck, 2016; Warburton et al., 2006). Experimental paradigms like Cyberball, a virtual ball-tossing game designed to induce feelings of exclusion, have demonstrated that even brief experiences of exclusion can trigger significant behavioral changes (Williams and Jarvis, 2006). The effects appear to be particularly pronounced when exclusion occurs in contexts relevant to one’s social identity or when it threatens fundamental needs for belonging and self-esteem (Riva and Eck, 2016). Beyond these immediate reactions, long-term social exclusion can solidify aggressive tendencies and poor self-regulation, beginning in early childhood and potentially creating persistent patterns of maladaptive behavior (Stenseng et al., 2014, 2015). This developmental trajectory suggests that early intervention targeting social acceptance may be critical for preventing the establishment of antisocial behavioral patterns (Arseneault et al., 2010; Crick and Dodge, 1994; Frick and Viding, 2009; Timeo et al., 2019).

These insights into social exclusion and antisocial behavior have important implications for understanding problematic behaviors in educational contexts. However, despite extensive research documenting the relationship between exclusion and behavioral outcomes, a critical gap remains in identifying the precise mechanisms and contextual factors that determine differential responses to social exclusion. While laboratory studies have demonstrated both prosocial and antisocial reactions to exclusion, the specific conditions that lead to one response versus another remain poorly understood. Particularly important is the question of how pre-existing social status within naturally occurring peer groups might moderate responses to acute exclusion experiences. Furthermore, most existing studies have examined general aggressive tendencies rather than the specific decision-making processes involved in antisocial risk-taking, which may be more representative of the calculated nature of, e.g., bullying behaviors observed in educational settings.

When examining the processes underlying behavioral responses to exclusion, decision-making mechanisms play a central role in determining whether individuals respond adaptively or maladaptively. According to the Social Information Processing (SIP) model (Crick and Dodge, 1994), individuals process social situations through several cognitive steps including encoding social cues, interpreting these cues, selecting goals, generating responses, evaluating responses, and enacting behavior. In this study, we focus particularly on how social exclusion may disrupt the goal selection and evaluation stage of this process, where individuals assess potential consequences of their actions for themselves and others.

As such, social exclusion can disrupt decision-making processes in multiple ways, leading to various behavioral outcomes. The emotional distress following exclusion can impair executive functioning and self-regulation, reducing the ability to inhibit impulsive responses (Baumeister et al., 2002, 2005). The cognitive depletion hypothesis suggests that socially excluded individuals have a reduced ability to engage in controlled, reflective cognitive processing (Walasek et al., 2019). While this theory does not directly predict aggressive tendencies, the impaired reflection may reduce consideration of others’ welfare, allowing self-focused motivations to predominate in decision-making. This might explain why some individuals respond with aggressive behaviors following exclusion, particularly when pre-existing individual differences predispose them toward antisocial tendencies. In contrast, according to the need-threat model, when social belonging is threatened, some individuals become more attuned to reconnection opportunities, leading to more prosocial behaviors (Maner et al., 2007). For others, however, the frustration-aggression hypothesis suggests that exclusion triggers defensive cognitive biases favoring self-protection over social connection, particularly when reintegration seems impossible (Stenseng et al., 2014; Timeo et al., 2019).

These alterations in decision-making processes may be particularly concerning during adolescence when neural systems supporting impulse control are still developing (Casey, 2015). For adolescents who engage in bullying behaviors, these altered decision processes may manifest as antisocial risk-taking—making decisions that potentially benefit themselves while accepting or seeking harm to others (Do et al., 2017). Bullying represents a particularly relevant form of antisocial behavior in school settings, characterized by strategic actions that enhance personal status while disregarding potential harm to peers. Rather than exhibiting general deficits in decision-making, individuals with antisocial tendencies often demonstrate specific patterns of risk preferences, particularly in social contexts where their actions affect others.

Research examining decision-making in students with antisocial tendencies has yielded mixed findings. Some studies suggest that bullies have broad problems with decision-making (Medeiros et al., 2016), while others indicate that they demonstrate strategic risk-taking abilities that actually improve with age (Flouri and Papachristou, 2019). These inconsistencies may be explained by considering that antisocial individuals engage not in general risk-taking but in specific forms of social risk-taking that benefit themselves at others’ expense. Bullies often exhibit a tendency to maximize personal benefits while disregarding potential harm to others, suggesting a specific pattern of value-based decision-making (Flouri and Papachristou, 2019; Olthof et al., 2011; Reijntjes et al., 2013). This antisocial risk-taking framework helps explain the strategic, calculated nature of many bullying behaviors, particularly those aimed at establishing dominance or acquiring resources at others’ expense (Olthof et al., 2011; Peeters et al., 2010).

While the presented theoretical perspectives may appear contradictory, they can be integrated by considering both individual differences and contextual factors that influence responses to exclusion. Rather than viewing the social reconnection theory and frustration-aggression hypothesis as competing frameworks, we propose that they may operate simultaneously but manifest differently depending on individuals’ pre-existing social status and specific contextual factors. Well-integrated individuals may have the social resources and adaptive coping strategies to respond to exclusion with increased attention to social cues and prosocial behavior (consistent with social reconnection theory), while poorly integrated individuals may experience exclusion as confirming their marginalized status, triggering defensive responses characterized by frustration and aggression. By examining how both chronic (classroom social acceptance) and acute (experimentally induced) forms of social exclusion interact to influence antisocial risk-taking, we can develop a more nuanced understanding of the specific conditions under which exclusion leads to adaptive versus maladaptive behavioral responses.

Beyond social and cognitive factors, moral cognition also plays a role in antisocial behavior. Moral disengagement involves cognitive mechanisms that allow individuals to justify harmful actions by disengaging from their internalized moral standards (Bandura, 1999). These mechanisms include moral justification, euphemistic labeling, diffusion of responsibility, disregarding consequences, dehumanization, and attribution of blame. Through these processes, individuals can reframe harmful actions as acceptable, reducing anticipated guilt and self-censure. Research has consistently linked moral disengagement with various forms of antisocial conduct, including aggression and bullying (Killer et al., 2019; Mazzone et al., 2021; Obermann, 2013).

The relationship between moral disengagement and decision-making processes may be particularly relevant for understanding antisocial behavior in the context of social exclusion. Moral disengagement might facilitate antisocial risk-taking by neutralizing concerns about harm to others that would typically constrain such behaviors (Mazzone et al., 2019; Perren et al., 2012). Following exclusion experiences, individuals might be more likely to engage in moral disengagement to justify retaliatory actions, viewing their behavior as warranted by their prior mistreatment. This could lead to a pattern where social exclusion increases moral disengagement, which in turn enables antisocial behavior, though this potential pathway warrants further investigation.

Despite these established connections between moral disengagement and antisocial behavior, a significant research gap remains in understanding how social exclusion might influence moral disengagement processes, particularly in adolescents. While theoretical frameworks suggest that exclusion could increase moral disengagement by providing justifications for retaliatory antisocial behavior, this potential pathway has received limited empirical attention. Furthermore, researchers have rarely examined whether individual differences in classroom social integration might moderate this relationship, and even fewer studies have investigated these processes in controlled experimental settings. This represents a critical gap, as understanding the interplay between social exclusion, moral disengagement, and antisocial risk-taking could provide valuable insights into the cognitive mechanisms that enable some adolescents to engage in harmful behaviors despite typically internalized moral standards.

In this study, we aimed to clarify the links between adolescents’ classroom social acceptance and the influence of acute experimentally induced social exclusion on antisocial behavior. We specifically designed our methodology to address limitations in previous research by incorporating both naturalistic measures of classroom social acceptance and experimental manipulation of exclusion experiences. Our experimental approach consisted of three key phases. First, we assessed each participant’s level of classroom social acceptance using sociometric measures, providing an indicator of their established social status among peers. Second, we randomly assigned participants to experience either social inclusion or exclusion using the Cyberball paradigm (Williams and Jarvis, 2006), a virtual ball-tossing game that reliably induces feelings of exclusion. Finally, we measured antisocial risk-taking using an adapted version of the Columbia Card Task (Figner et al., 2009), modified to capture decisions that potentially benefited the participant while risking harm to others, as well as moral disengagement. This sequential design allowed us to examine how pre-existing social status (established classroom acceptance) interacts with acute experimentally-induced exclusion to influence antisocial tendencies.

The novelty of our approach lies in our examination of antisocial risk-taking specifically, rather than general aggression or nonspecific antisocial behavior. Based on the theoretical framework proposed by Do et al. (2017), we conceptualized antisocial risk-taking as behavior that involves pursuing personal benefits while accepting or seeking risks that harm others. This conceptualization aligns with characteristics observed in bullying behavior, which often involves calculated actions to enhance one’s own status or acquire resources at others’ expense (Peeters et al., 2010). By measuring decision-making in an antisocial context, we aimed to capture the cognitive processes that may underlie bullying tendencies.

**Figure 1:**
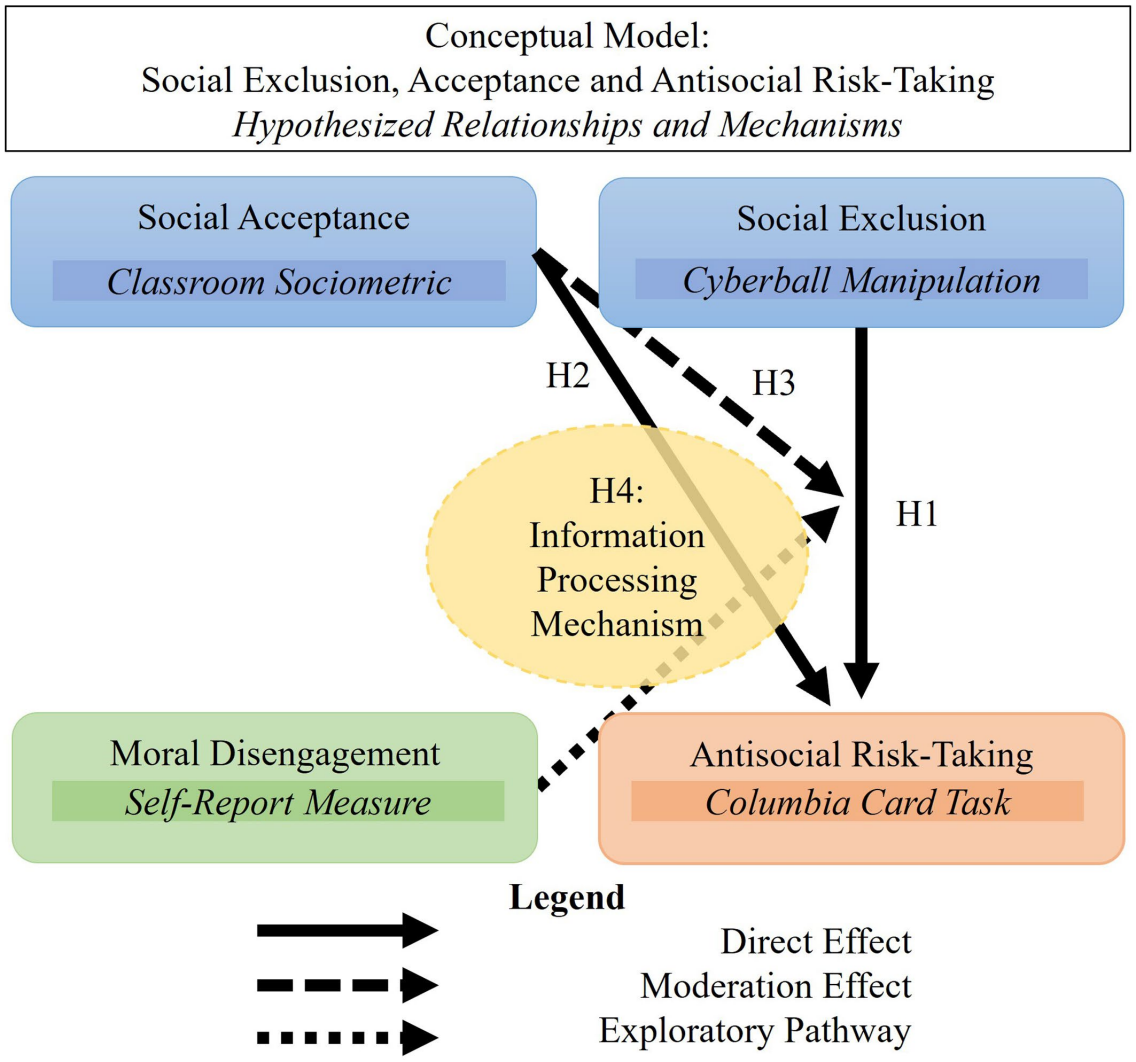
Schematic representation of the conceptual model and hypotheses.

We hypothesized that: (1) experimentally induced social exclusion would increase tendencies towards antisocial risk-taking, consistent with the frustration-aggression hypothesis suggesting that exclusion triggers antisocial responses; (2) adolescents with poor classroom social acceptance would show greater antisocial risk-taking, reflecting a potential cumulative effect of chronic social exclusion on behavioral tendencies; (3) adolescents with low classroom social acceptance would react more strongly to experimentally induced social exclusion with antisocial risk-taking, demonstrating an interactive effect whereby acute exclusion particularly affects those already experiencing chronic exclusion; and (4) adolescents with low classroom social acceptance would show reduced information processing in decision-making following experimentally induced exclusion, suggesting impaired consideration of relevant information when making decisions in antisocial contexts. Additionally, we explored whether moral disengagement relates to antisocial risk-taking, particularly in the context of experimentally induced social exclusion. While we did not formulate a formal hypothesis about this relationship due to the exploratory nature of this analysis, examining this potential connection might provide valuable insights into the cognitive mechanisms that enable antisocial behavior following exclusion experiences. The suggested conceptual model and hypotheses are visualized in Figure 1.

By integrating measures of classroom social acceptance, experimental manipulation of social exclusion, and a novel paradigm for assessing antisocial risk-taking, this study provides new insights into the mechanisms and factors that may predispose adolescents to antisocial behavior in response to social exclusion experiences. Understanding these relationships has important implications for educational practice, potentially informing more targeted intervention approaches for preventing bullying and other forms of antisocial behavior in school settings.

## 2 Materials and methods

### 2.1 Participants

For this study, 141 adolescents were recruited from 7 classes in years 7–9 of the German school system, located in the vicinity of the University of Wuppertal. Participants who did not engage meaningfully with the task were excluded (*n* = 12).^1^ As shown in Table 1 in more detail, the final sample consisted of 129 adolescents from age 12 to 16.

**Table 1:** Demographic characteristics and descriptive statistics by school class and social condition.

Class (n)	Condition	N	Age	% Female	Cards	Acceptance
7th (2)	Exclusion	19	12.95 (0.62)	47.4	7.11 (3.64)	1.81 (0.61)
	Inclusion	19	12.68 (0.67)	57.9	8.17 (3.11)	1.91 (0.46)
8th (3)	Exclusion	26	13.73 (0.53)	46.2	6.28(3.86)	2.14 (0.49)
	Inclusion	26	13.62 (0.64)	34.6	7.55 (5.15)	1.95 (0.58)
9th (2)	Exclusion	19	14.05 (0.4)	47.4	5.91 (2.3)	1.88 (0.38)
	Inclusion	20	14.3 (0.57)	55.0	5.99 (3.31)	1.96 (0.55)
Total (7)	Exclusion	64	13.59 (0.68)	47.6	6.42 (3.38)	1.97 (0.51)
	Inclusion	65	13.55 (0.88)	49.2	7.25 (4.14)	1.94 (0.53)
	Combined	129	13.57 (0.79)	47.3	6.84 (3.79)	1.95 (0.52)

Age is reported in years. Cards refers to the mean number of cards drawn in the antisocial risk-taking task. Condition refers to the experimentally induced social inclusion or exclusion condition and Acceptance to the classroom social acceptance score. Participants were randomly assigned to either inclusion or exclusion condition.

Due to technical problems, moral disengagement was only assessed in some of the classes tested for the study, resulting in a smaller subsample of participants who also completed the moral disengagement scale (*n* = 82). Therefore, the effects of moral disengagement that were also to be explored were tested in a separate model in this smaller subsample. The subsample is described in more detail in Table 2. The subsample did not differ from the full sample on relevant variables such as overall classroom acceptance (*t*(203) = −0.53, *p* = 0.60) or antisocial risk-taking (*t*(203) = −0.01, *p* = 0.99).

**Table 2:** Demographic characteristics and descriptive statistics by school class and social condition of the subsample of participants that also filled in the moral disengagement scale.

Class (n)	Condition	N	Age	% Female	Cards	Acceptance	Disengagement
7th (1)	Exclusion	8	12.62 (0.52)	62.5	6.91 (3.20)	2.17 (0.6)	0.59 (0.19)
	Inclusion	6	12.67 (0.52)	50.0	8.22 (2.37)	2.27 (0.34)	0.73 (0.26)
8th (2)	Exclusion	14	13.57 (0.65)	42.9	6.34 (4.08)	2.13 (0.42)	0.65 (0.48)
	Inclusion	15	13.27 (0.46)	40.0	9.89 (5.53)	1.85 (0.62)	0.94 (0.59)
9th (2)	Exclusion	19	14.05 (0.4)	47.4	5.91 (2.30)	1.88 (0.38)	0.69 (0.37)
	Inclusion	20	14.3 (0.57)	55.0	5.99 (3.31)	1.96 (0.55)	0.81 (0.49)
Total (5)	Exclusion	41	13.61 (0.74)	48.8	6.25 (3.12)	2.02 (0.45)	0.66 (0.38)
	Inclusion	41	13.68 (0.82)	48.8	7.74 (4.46)	1.97 (0.55)	0.81 (0.49)
	Combined	82	13.65 (0.78)	48.8	7.00 (3.90)	1.99 (0.51)	0.75 (0.45)

Age is reported in years. Cards refers to the mean number of cards drawn in the antisocial risk-taking task. Condition refers to the experimentally induced social inclusion or exclusion condition and Acceptance to the classroom social acceptance score. Disengagement refers to the Moral Disengagement score. Participants were randomly assigned to either inclusion or exclusion condition.

All participants provided informed consent and were fully debriefed upon completion of the study. The debriefing protocol included explanation of the study’s objectives, clarification that virtual peers in the experimental tasks were computer-controlled, and discussion of the social exclusion manipulation. This procedure was approved as part of the institutional ethical review process.

### 2.2 Materials and procedure

The study employed a sequential experimental design conducted across both classroom and individual settings.

#### 2.2.1 Phase 1: classroom assessment

Initially, all participants completed the Social Acceptance Measure during a regular class period. To measure students’ classroom social acceptance two sociometric questionnaires were used (Moreno, 1953). Sociometric methods are commonly used in educational research to study how students in a classroom relate to each other (Cillessen, 2009). Therefore, each student in a classroom evaluates all other students in regard to a specific criterion in a round-robin design. To account for possible differences when choosing others for inside or outside classroom activities, the present study used two different criteria: Each participant rated for each student in their class on a five-point Likert scale (0 = not very much, 4 = very much) how much they would like to (1) spend time with the student during the break and (2) work with the student in class. Ratings received for both criteria were averaged for each participant. Due to the high correlation (*r* = 0.922, *p* < 0.001) between the two sociometric criteria, an average score was calculated for each participant.

#### 2.2.2 Phase 2: individual experimental sessions

Following the classroom assessment, participants proceeded to individual experimental sessions conducted in separate rooms. Participants were seated in front of tablets executing the tasks and questionnaires individually but with up to five students being present in the room. Participants were informed that they would be connected to peers in other rooms via an internal network for collaborative tasks. All experimental paradigms were administered on tablets in the following sequence:

1 Virtual peer introduction and Cyberball paradigm

The experimental procedure for inducing social exclusion, described below, follows the methodology established in our previous work (Lorenz and Ferdinand, 2025), utilizing identical manipulation protocols and visual materials. To create a realistic social interaction context, participants were ostensibly connected with two virtual peers through a simulated network interface. Participants were informed that these virtual peers were other students participating simultaneously from separate rooms. Each participant developed a personal profile including a chosen avatar, one leisure activity, and demographic information. These profiles, along with pre-constructed profiles of the two virtual peers, were displayed to all participants to establish a sense of social connection.

Following this introduction phase, participants engaged in a virtual ball-tossing activity designed to manipulate feelings of social inclusion or exclusion, based on the established paradigm by Williams and Jarvis (2006). Participants were instructed to practice their mental visualization abilities by imagining themselves actively participating in the game environment while interacting with the two fictitious peers they had previously been introduced to. Participants used the left and right arrow keys to pass the ball to the respective confederates when in possession. All players were represented by their previously chosen avatars throughout the game.

The experimental manipulation varied the frequency of ball reception: In the inclusion condition, participants received the ball approximately one-third of the time (equal to other players), while in the exclusion condition, participants received the ball significantly less often—only 5 times out of 30 total tosses (16%). Participants were randomly assigned to the inclusion and exclusion conditions. This methodology allowed for examination of how social exclusion experiences might influence subsequent antisocial risk-taking behaviors.

2 Antisocial risk-taking task

Immediately following the Cyberball experience, participants completed an adapted version of the Columbia Card Task (CCT, Figner et al., 2009) to evaluate participants’ willingness to engage in antisocial risk-taking. The task presented participants with a grid of 32 face-down cards arranged in 4 rows. Each card, when selected, revealed either a smiley face (representing gains) or a frowny face (representing losses). Participants chose how many cards to turn over in each trial, with higher numbers of cards indicating higher levels of antisocial risk-taking, as the probability for positive outcomes for oneself increases but also the chance for others to lose a relatively large number of points.

Participants received feedback about each trials’ outcome after their selection. That is, unlike the original “hot” version of the CCT, trials continued even after encountering loss cards (non-censored approach). Each trial displayed key information at the right side of the screen: points gained per positive card (either 10 or 30 points), points lost per negative card (either 250 or 750 points), and the number of loss cards present in the deck (either 1 or 3).

**Figure 2:**
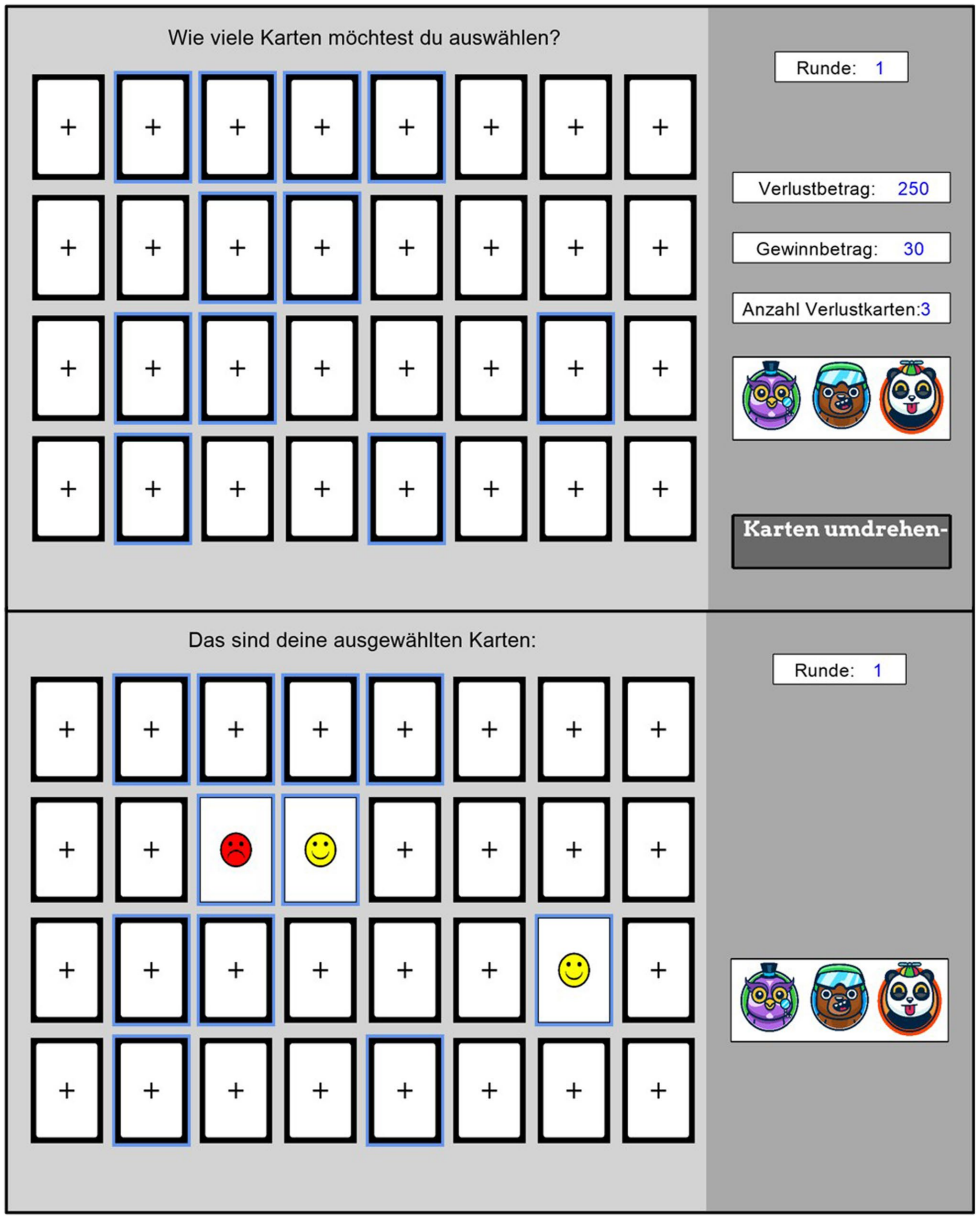
Antisocial Columbia Card Task. The adapted Columbia Card Task showing the decision interface (upper panel) asking participants to draw as many cards as they like (“Wie viele Karten möchtest du auswählen?”) and feedback screen (lower panel) showing the face sides of the previously chosen cards (“Das sind deine ausgewählten Karten”). Participants select how many cards to turn over, with the current round number (“Runde”) and task parameters displayed on the right (loss amount [“Verlustbetrag”], gain amount [“Gewinnbetrag”], and number of loss cards [“Anzahl Verlustkarten”]). The key adaptation is shown by the player avatars with a red circle indicating which virtual peer will lose points, transforming this into an antisocial risk-taking paradigm where participants gain points for themselves while potentially causing losses for others.

Moreover, as we adapted the task to represent antisocial risk-taking (see Do et al., 2017 for a definition), the avatars of all three players appeared alongside the card display. Participants were informed that any gained points would be added to their personal balance, while a red circle around one of the virtual peers’ avatars indicated that any losses would be deducted from that peer’s account. This visual indicator was consistently displayed on the right side of the screen. After each selection, participants received feedback showing how many gain points they accumulated (see Figure 2).

Participants completed a total of 32 trials. The experimental design included all possible combinations of three factors: gain amounts (10 or 30 points), loss amounts (250 or 750 points), and number of loss cards (1 or 3), creating 8 unique trial types (2 × 2 × 2). Each of these 8 trial types was presented 4 times across the 32 trials. Importantly, for half the trials (16 trials), one virtual confederate was designated to receive any losses (indicated by a red circle around their avatar), while for the other half (16 trials), the second confederate was the designated recipient of losses.

3 Moral disengagement scale

Finally, participants completed the Unified Measure of Moral Neutralization Questionnaire (Ribeaud and Eisner, 2010) to assess moral disengagement after the antisocial risk-taking task. This sequence was deliberately chosen to avoid potentially influencing participants’ behavior in the risk-taking task through moral-related questions that might have prompted self-reflection or heightened awareness of moral considerations. While the temporal sequence of measurement places moral disengagement after the behavioral assessment, we conceptualize moral disengagement as a relatively stable cognitive tendency that likely influenced behavior during the task. This approach allows us to explore whether individual differences in moral disengagement might help explain variance in antisocial risk-taking, while acknowledging that the experimental manipulation may have also influenced reported moral disengagement levels. The scale consists of 18 items that reflect different mechanisms to justify deviant behavior (e.g., “Some young people are teased because they deserve it”). Responses were given on a 4-point Likert scale (0 = fully untrue; 3 = fully true) and averaged for each participant (*ω* = 0.880).

This methodological approach enabled us to investigate how induced social exclusion experiences affect antisocial risk-taking behaviors, while examining how these effects might be moderated by participants’ classroom social acceptance status.

### 2.3 Statistical analyses

#### 2.3.1 Linear mixed effect models

We analyzed the data using generalized linear mixed-effects models (GLMMs) with a Poisson distribution to account for the count nature of our dependent variable (number of cards clicked). The full model included classroom social acceptance (mean-centered continuous variable), social exclusion condition (sum-coded as −1/1), and task information variables (gain amount, loss amount, and loss cards, all sum-coded as −1/1) as fixed effects, including all possible interactions up to the three-way level between classroom social acceptance, experimentally induced social exclusion, and each task information variable.

Following recommendations by Barr et al. (2013), we initially specified a maximal random effects structure:


$$\begin{array}{c} \log(Y_{\text{ijkl}})=\beta_{0}+\beta_{1}{\mathrm{SA}}_{i}+\beta_{2}{\mathrm{SE}}_{j}+\beta_{3}{\mathrm{GA}}_{k}+\beta_{4}{\mathrm{LA}}_{k}+\beta_{5}{\mathrm{LC}}_{k}\\ +\beta_{6}({\mathrm{SA}}_{i}\;x\;{\mathrm{SE}}_{j})+\beta_{7}({\mathrm{SA}}_{i}\;x\;{\mathrm{GA}}_{k})+\beta_{8}({\mathrm{SA}}_{i}\;x\;{\mathrm{LA}}_{k})\\ +\beta_{9}({\mathrm{SA}}_{i}\;x\;{\mathrm{LC}}_{k})+\beta_{10}({\mathrm{SE}}_{j}\;x\;{\mathrm{GA}}_{k})\\ +\beta_{11}\;({\mathrm{SE}}_{j}\;x\;{\mathrm{LA}}_{k})+\beta_{12}({\mathrm{SE}}_{j}\;x\;{\mathrm{LC}}_{k})\\ +\beta_{13}({\mathrm{SA}}_{i}\;x\;{\mathrm{SE}}_{j}\;x\;{\mathrm{GA}}_{k})+\beta_{14}({\mathrm{SA}}_{i}\;x\;{\mathrm{SE}}_{j}\;x\;{\mathrm{LA}}_{k})\\ +\beta_{14}({\mathrm{SA}}_{i}\;x\;{\mathrm{SE}}_{j}\;x\;{\mathrm{LC}}_{k})+u_{0i}+u_{1i}{\mathrm{GA}}_{k}+u_{2i}{\mathrm{LA}}_{k}\\ +u_{3i}{\mathrm{LC}}_{k}+v_{0l}+\varepsilon_{\mathrm{jkl}.} \end{array}$$

where $Y_{\text{ijkl}}$ is the expected count of cards clicked, ${\mathrm{SA}}_{i}\;$ is the mean-centered classroom social acceptance score, ${\mathrm{SE}}_{j}$ is the social exclusion condition (sum-coded), ${\mathrm{GA}}_{k}$ , ${\mathrm{LA}}_{k}$ , and ${\mathrm{LC}}_{k}$ are the sum-coded task information variables (gain amount, loss amount, and loss cards), $u_{0i}$ is the random intercept for participant i, $u_{1i}$ , $u_{2i}$ , and $u_{3i}$ are the random slopes for the task information variables for participant i, $v_{0l}$ is the random intercept for class l, and $\varepsilon_{\mathrm{jkl}}$ is the residual error term.

After confirming model convergence, we examined the variance components and correlation structure of the random effects. The analysis revealed substantial variance explained by participant-level (*SD* = 0.48) and class-level random intercepts (*SD* = 0.16), but considerably smaller variation in the random slopes for gain amount (*SD* = 0.08), loss amount (*SD* = 0.07), and loss cards (*SD* = 0.09). Correlations between these random effects were generally weak (all |r| ≤ 0.25), with correlations between intercept and random slopes ranging from −0.03 to 0.05, and correlations between slopes ranging from −0.01 to 0.25, suggesting limited dependency between individual differences in these effects.

Based on this pattern, we adopted a more parsimonious model with random intercepts only:


$$\begin{array}{c} \log(Y_{\text{ijkl}})=\beta_{0}+\beta_{1}{\mathrm{SA}}_{i}+\beta_{2}{\mathrm{SE}}_{j}+\beta_{3}{\mathrm{GA}}_{k}+\beta_{4}{\mathrm{LA}}_{k}+\beta_{5}{\mathrm{LC}}_{k}\\ +\beta_{6}({\mathrm{SA}}_{i}\;x\;{\mathrm{SE}}_{j})+\beta_{7}({\mathrm{SA}}_{i}\;x\;{\mathrm{GA}}_{k})+\beta_{8}({\mathrm{SA}}_{i}\;x\;{\mathrm{LA}}_{k})\\ +\beta_{9}({\mathrm{SA}}_{i}\;x\;{\mathrm{LC}}_{k})+\beta_{10}({\mathrm{SE}}_{j}\;x\;{\mathrm{GA}}_{k})\\ +\beta_{11}\;({\mathrm{SE}}_{j}\;x\;{\mathrm{LA}}_{k})+\beta_{12}({\mathrm{SE}}_{j}\;x\;{\mathrm{LC}}_{k})\\ +\beta_{13}({\mathrm{SA}}_{i}\;x\;{\mathrm{SE}}_{j}\;x\;{\mathrm{GA}}_{k})+\beta_{14}({\mathrm{SA}}_{i}\;x\;{\mathrm{SE}}_{j}\;x\;{\mathrm{LA}}_{k})\\ +\beta_{14}({\mathrm{SA}}_{i}\;x\;{\mathrm{SE}}_{j}\;x\;{\mathrm{LC}}_{k})+u_{0i}+v_{0l}+\varepsilon_{\mathrm{jkl}} \end{array}$$

This approach balanced our goals of capturing meaningful individual differences while maintaining model parsimony. The same random structure was used for the model of the exploratory analysis adding moral disengagement as a predictor of antisocial risk-taking in the reduced sample. All analyses were conducted using R (version 2023.06.1, R Core Team, 2023). Generalized linear mixed-effects models were fitted using the lme4 package (Bates et al., 2015), *p*-values were calculated via Satterthwaite approximation using the lmerTest package (Kuznetsova et al., 2017), and the emmeans package was used for simple effects testing (Lenth, 2025). This study was not pre-registered. Data and analysis code will be made publicly available upon publication via the Open Science Framework (OSF, https://osf.io/gp8uk/).

#### 2.3.2 Statistical power

We conducted an a-priori power analysis using GPower 3.1 (Faul et al., 2009) to estimate sample size requirements. Since GPower does not directly support power calculations for linear mixed effect models with continuous predictors, we approximated by dichotomizing the continuous classroom social acceptance variable. We used the test for ANOVAs with between-within interactions with parameters for both medium (*f* = 0.25) and small (*f* = 0.15) effect sizes, alpha = 0.05, power = 0.80, number of groups = 4 (for power calculation purposes only), number of measurements = 2 (representing the two levels of task information variables such as gain amount, loss amount, or loss probability), correlation among repeated measures = 0.5, and non-sphericity correction = 1. For a medium effect size, this yielded a required total sample size of 48 participants (*λ* = 12.00, critical *F* = 2.82, *df* = 3, 44), corresponding to 24 participants per social exclusion condition. For a small effect size, 128 participants were required (*λ* = 11.52, critical *F* = 2.68, *df* = 3, 124), or 64 participants per social exclusion condition. In the actual analysis, classroom social acceptance was used as a continuous predictor and random effects at the participant and class-level were included, which ultimately increased the power to detect effects.

## 3 Results

Overall, adolescents drew *M* = 6.84 (*SD* = 3.79, min = 1.28, max = 20.59) cards and their classroom social acceptance score was *M* = 1.95 (*SD* = 0.52) on average (see also Table 1). To examine whether participants’ baseline classroom social acceptance was similar in both groups, we compared classroom social acceptance scores between the groups experiencing either social inclusion or exclusion. Results showed no significant difference between the inclusion (*M* = 1.94, *SD* = 0.53) and exclusion conditions (*M* = 1.97, *SD* = 0.51), *t*(127) = 0.27, *p* = 0.79, *d* = 0.05, *% CI* [−0.30, 0.39]. This indicates that participants in both Cyberball condition groups did not differ in the reported levels of classroom social acceptance on average.

Table 3 shows the results of the linear mixed model of the effects of individual classroom social acceptance (SA), social exclusion (SE), and task information on antisocial risk-taking.

**Table 3:** Model estimation results for the full sample.

Predictors	Estimates	Conf. Int (95%)	*p*-value
(Intercept)	1.80	1.66–1.95	<0.001
Acceptance	−0.07	−0.25–0.11	0.458
Condition [Inclusion vs. Exclusion]	−0.05	−0.14–0.04	0.269
Gain Amount [10 vs 30 points]	−0.02	−0.03–−0.00	0.009
Loss Amount [250 vs. 750 points]	-0.00	−0.01–0.01	0.828
Loss Cards [1 vs. 3 cards]	−0.01	−0.02–0.00	0.171
Acceptance x Condition	−0.04	−0.22–0.13	0.643
Acceptance x Gain Amount	0.00	−0.02–0.03	0.769
Acceptance x Loss Amount	−0.00	−0.02–0.02	0.878
Acceptance x Loss Cards	0.04	0.02–0.06	<0.001
Condition x Gain Amount	−0.00	−0.01–0.01	0.726
Condition x Loss Amount	−0.00	−0.02–0.01	0.430
Condition x Loss Cards	0.01	−0.01–0.02	0.392
Acceptance x Condition x Gain Amount	−0.01	−0.03–0.01	0.330
Acceptance x Condition x Loss Amount	−0.01	−0.03–0.02	0.624
Acceptance x Condition x Loss Cards	0.03	0.01–0.05	0.010
Random effects			
σ₂	0.13		
τ participant	0.26		
τ class	0.02		
ICC	0.68		
N participant	129		
N class	7		
Observations	4,128		
Marginal R2 / Conditional R2	0.013 / 0.683		

Model estimation results for number of cards representing the tendency to engage in antisocial risk behavior including predictors, estimates, and significance.

### 3.1 Hypothesis 1: effects of experimentally induced social exclusion

Contrary to our first hypothesis, we did not find a significant main effect of social exclusion on antisocial risk-taking behavior (*Est.* = − 0.05, *SE =* 0.05, *p = 0*.269), suggesting that experimentally induced social exclusion alone did not increase tendencies towards antisocial risk-taking.

### 3.2 Hypothesis 2: classroom social acceptance

The second hypothesis predicted that adolescents with low classroom social acceptance would show greater antisocial risk-taking. While we observed a consistent negative slope for classroom social acceptance across most conditions (*Est.* = − 0.07, *SE =* 0.09, *p =* 0.458), these effects did not significantly differ from zero (all *p*s > 0.092).

### 3.3 Hypothesis 3: interaction between classroom social acceptance and experimentally induced social exclusion

**Figure 3:**
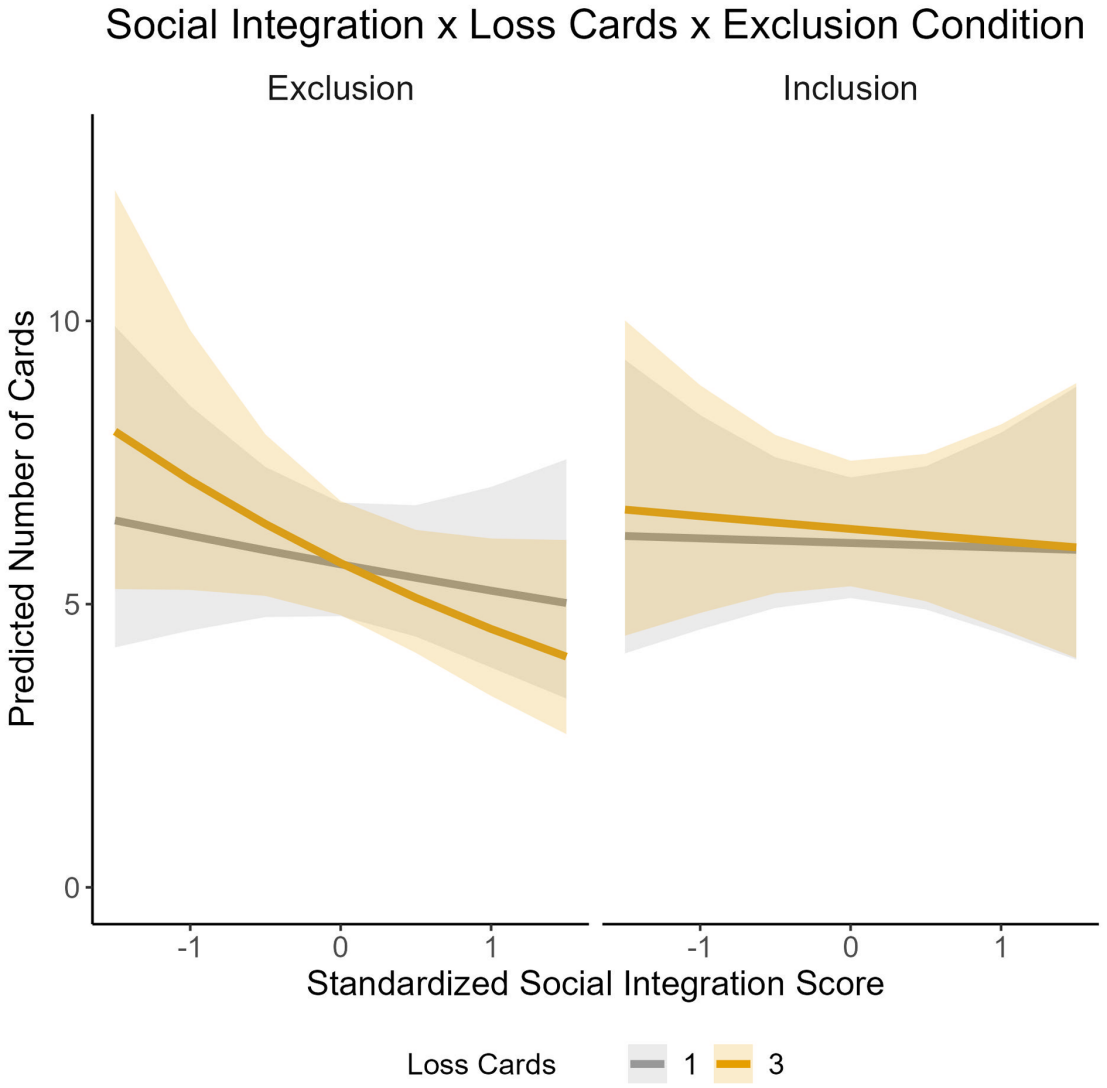
Interaction effect between the standardized classroom social acceptance score, loss cards (1 vs. 3), and social exclusion condition (Inclusion vs. Exclusion) on the predicted number of cards selected. shaded areas represent 95% confidence intervals.

Our third hypothesis suggested that adolescents with low classroom social acceptance would react more strongly with antisocial risk-taking to experimentally induced social exclusion. The results revealed a significant three-way interaction between classroom social acceptance, experimentally induced social exclusion, and loss cards (*Est.* = 0.03, *SE* = 0.01, *p* = 0.010), indicating a complex relationship between these factors (see Figure 3). Notably, we did not observe significant interactions involving loss amount (250 vs. 750 points). Neither the three-way interaction between classroom social acceptance, social exclusion, and loss amount (*p* = 0.624) nor the two-way interaction between classroom social acceptance and loss amount (*p* = 0.878) reached significance, suggesting that the magnitude of potential losses to others did not moderate responses to exclusion in the same way as loss probability.

Breaking down this interaction, we found distinct patterns in how loss cards affected behavior across different levels of classroom social acceptance in inclusion versus exclusion conditions. In the exclusion condition, participants with high classroom social acceptance scores (+1 *SD*) showed significantly fewer card selections when the number of loss cards was high (3 loss cards) compared to low (1 loss card) (*Est.* = 0.13*, SE* = 0.04, *z* = 3.52, *p* = <0.001). Conversely, participants with low classroom social acceptance scores (−1 SD) in the exclusion condition showed the opposite pattern, selecting more cards with a high as compared to low number of loss cards (*Est.* = − 0.14, *SE* = 0.04, *z* = −3.90*, p* = < 0.001). This provides partial support for our hypothesis, as adolescents with low classroom social acceptance did indeed show maladaptive responses when socially excluded in conditions with higher loss probability.

### 3.4 Hypothesis 4: information processing in antisocial risk-taking

Our fourth hypothesis predicted that adolescents with low classroom social acceptance would show reduced information use in antisocial risk-taking, especially after social exclusion. The significant interaction between classroom social acceptance and loss cards (*Est.* = 0.04, *SE* = 0.01, *p* < 0.001) and the three-way interaction with social exclusion suggest that information use about loss probabilities indeed was influenced by social exclusion versus inclusion.

However, contrary to reduced information processing, individuals with high and low scores in classroom social integration showed a greater adjustment to loss probabilities in the social exclusion group than individuals with scores in the middle of the scale. Participants with high classroom social integration scores (+1 *SD*) selected significantly fewer cards when the number of loss cards was high (3 loss cards) compared to low (1 loss card). Conversely, participants with low classroom social integration scores (−1 *SD*) selected significantly more cards when the number of loss cards was high compared to low. Participants with middle-range classroom social acceptance scores showed smaller differences in card selections between high and low loss probability conditions.

In the inclusion condition, the pattern was different: only participants with intermediate classroom social acceptance scores selected more cards when the number of loss cards was high as compared to low (*Est.* = −0.03, *SE* = 0.02, *z* = −1.63, *p* = 0.103). No significant differences were found for high (*p* = 0.191) and low classroom social acceptance scores (*p* = 0.850).

There also was a main effect of gain amount (*Est.* = − 0.02, *SE* = 0.01, *p* = 0.009), indicating that participants showed more card selections when gain amounts were larger (10 vs. 30 points). This suggests that while information processing about gains remained similar across groups, processing of risk information (loss cards) varied significantly based on classroom social acceptance and exclusion.

Several other interactions did not reach significance, including the two-way interaction between classroom social acceptance and exclusion condition (*p* = 0.436), and three-way interactions involving gain amount (*p* = 0.208). Additionally, main effects for loss amount (*p* = 0.758) and loss cards (*p* = 0.074) were not significant.

### 3.5 Exploratory analysis: moral disengagement and antisocial risk-taking

The moral disengagement score was *M* = 0.75 (*SD* = 0.45) on average. To examine whether participants’ average moral disengagement was similar in both groups, we compared moral disengagement scores between the groups experiencing either social inclusion or exclusion. Results showed a marginally not significant difference with a moderate effect size between the inclusion (*M* = 0.85, *SD* = 0.50) and exclusion groups (*M* = 0.66, *SD* = 0.38), *t*(80) = −1.93, *p* = 0.06, *d* = −0.43, % CI [−0.86, 0.01]. This indicates a pattern that warrants consideration in our discussion despite the borderline statistical significance.

As part of our exploratory analyses, we examined whether moral disengagement, measured after the experimental task, was associated with antisocial risk-taking behavior. For the full pattern of results see Table 4. Our analysis revealed a significant main effect of moral disengagement on antisocial risk-taking (*Est.* = 0.36, *SE* = 0.14, *p* = 0.009), with a positive estimate indicating that higher moral disengagement was associated with increased antisocial risk-taking behavior. We did not find significant interactions between moral disengagement and social exclusion (*Est.* = 0.11, *SE* = 0.14, *p* = 0.436), suggesting that the relationship between moral disengagement and antisocial risk-taking remained relatively consistent regardless of whether adolescents experienced social inclusion or exclusion.

**Table 4:** Model estimation results for the subsample of participants that also filled in the Moral Disengagement Scale.

Predictors	Estimates	Conf. Int (95%)	*p*-value
(Intercept)	1.81	1.67–1.95	<0.001
Disengagement	0.36	0.09–0.63	0.009
Acceptance	0.25	−0.01–0.51	0.063
Condition [Inclusion vs. Exclusion]	−0.07	−0.18–0.04	0.240
Gain Amount [10 vs. 30 points]	−0.01	−0.02–0.01	0.287
Loss Amount [250 vs 750 points]	−0.02	−0.03–0.00	0.034
Loss Cards [1 vs. 3 cards]	0.00	−0.01–0.02	0.518
Acceptance x Disengagement	0.11	−0.16–0.38	0.436
Acceptance x Condition	0.09	−0.15–0.32	0.476
Disengagement x Gain Amount	0.05	0.01–0.09	0.006
Disengagement x Loss Amount	0.03	−0.01–0.07	0.100
Disengagement x Loss Cards	−0.03	−0.06–0.01	0.115
Acceptance x Gain Amount	0.00	−0.03–0.03	0.900
Acceptance x Loss Amount	0.03	−0.00–0.06	0.082
Acceptance x Loss Cards	−0.00	−0.03–0.03	0.892
Condition x Gain Amount	0.01	−0.00–0.03	0.149
Condition x Loss Amount	−0.01	−0.02–0.01	0.413
Condition x Loss Cards	0.01	0.00–0.03	0.023
Disengagement x Condition x Gain Amount	0.01	−0.03–0.05	0.573
Disengagement x Condition x Loss Amount	0.00	−0.03–0.04	0.881
Disengagement x Condition x Loss Cards	0.01	−0.03–0.05	0.593
Acceptance x Condition x Gain Amount	−0.02	−0.05–0.01	0.296
Acceptance x Condition x Loss Amount	−0.01	−0.04–0.02	0.524
Acceptance x Condition x Loss Cards	0.00	−0.03–0.03	0.862
Random effects			
σ₂	0.13		
τ participant	0.24		
τ class	0.01		
ICC	0.65		
N participant	82		
N class	5		
Observations	2,624		
Marginal R2/Conditional R2	0.085/0.676		

Model estimation results for number of cards representing the tendency to engage in antisocial risk behavior including predictors, estimates, and significance.

Additionally, a significant interaction between gain amount and moral disengagement was observed (*Est.* = 0.05, *SE* = 0.02, *p* = 0.006). This interaction suggests that while adolescents with higher moral disengagement after the experimental task generally took more antisocial risks, this effect was slightly attenuated when potential gains were larger. In other words, the influence of moral disengagement on antisocial risk-taking was more pronounced when the potential personal rewards were smaller.

Beyond our exploratory analysis of moral disengagement’s influence on antisocial risk-taking, we also examined the relationship between classroom social acceptance and moral disengagement. While this analysis was not part of our original hypotheses, it provides valuable insights into the association between these constructs.

Interestingly, we observed a significant negative correlation between moral disengagement and classroom social acceptance scores for the total sample (*r* = −0.34, *p* = <0.001), suggesting that adolescents who were less socially integrated tended to report higher levels of moral disengagement. When examining this relationship within the experimental groups, the correlation was significant in the exclusion condition group (*r* = −0.37, *p* = 0.020), while it did not reach significance in the inclusion condition (*r* = −0.31, *p* = 0.050).

## 4 Discussion

Our study investigated the relationship between social exclusion, classroom social acceptance, and antisocial risk-taking behavior among adolescents. The findings reveal a complex interplay between these factors, challenging simplistic views of how social exclusion influences antisocial tendencies. Contrary to our first hypothesis, experimentally induced social exclusion did not directly increase antisocial risk-taking; instead, responses depended significantly on adolescents’ pre-existing classroom social acceptance. We found a critical interaction between classroom social acceptance, social exclusion, and loss probability, indicating that well-accepted adolescents responded to exclusion by becoming more sensitive to risk information—reducing risky choices when loss probability was high for others—whereas adolescents with low classroom acceptance demonstrated the opposite pattern, increasing risky choices despite higher potential costs for others when excluded. Additionally, our exploratory analysis revealed that moral disengagement measured after the risk task significantly predicted antisocial risk-taking and was negatively correlated with classroom social acceptance. However, adolescents remained sensitive to potential rewards for themselves regardless of classroom social acceptance status or exclusion experiences.

### 4.1 Differential responses to social exclusion

The absence of a main effect for social exclusion suggests that responses to exclusion are more nuanced than previously theorized, depending heavily on individual differences and contextual factors. Importantly, this pattern should not be interpreted as a failure of our experimental manipulation, but rather as a theoretically meaningful finding that challenges simplistic interpretations of social exclusion effects. Rather than supporting a universal” social exclusion leads to aggression” narrative, our results demonstrate that responses to acute social exclusion are fundamentally contingent upon individual differences in classroom social acceptance status. This finding can help reconcile conflicting results in the social exclusion literature, where some studies report increased antisocial behavior following exclusion (Twenge et al., 2001; Warburton et al., 2006) while others observe more prosocial tendencies (Maner et al., 2007). Specifically, well-accepted adolescents showed adaptive responses to exclusion, becoming more cautious when potential losses for others were high, while poorly accepted adolescents demonstrated the opposite pattern. This aligns with the “outcast-lash-out effect” where social rejection triggers antisocial tendencies among marginalized individuals (Reijntjes et al., 2010; Warburton et al., 2006).

The role of classroom social acceptance as a moderator of exclusion effects provides important insights into why some adolescents develop antisocial tendencies while others do not. Rather than viewing antisocial responses as direct consequences of exclusion, our findings suggest a more complex pathway where pre-existing social vulnerabilities create differential susceptibility to exclusion experiences. For well-accepted adolescents, occasional experiences of exclusion may represent manageable social challenges that activate protective responses, while for poorly accepted adolescents, these same experiences may exacerbate existing feelings of rejection, triggering maladaptive coping strategies.

### 4.2 Information processing in antisocial risk-taking

In adapting the Columbia Card Task (CCT) for our study, we created the first variant specifically designed to capture antisocial risk-taking, where participants could gain points for themselves while risking losses for others. In the original CCT paradigm, participants typically show sensitivity to all decision parameters—increasing risky choices with larger gain amounts, smaller loss amounts, and lower loss probability (Figner et al., 2009; Lorenz and Ferdinand, 2025). However, our antisocial version revealed a more selective pattern of information use which provides important insights into the cognitive mechanisms underlying antisocial behavior.

While all adolescents remained consistently sensitive to information signaling potential rewards for themselves (gain amount) regardless of the social exclusion manipulation, their processing of information concerning risk for others (loss probability) varied significantly based on classroom social acceptance and exclusion condition. Interestingly, while loss probability (number of loss cards) significantly moderated responses to exclusion, loss magnitude (250 vs. 750 points) did not. This pattern aligns with established findings from the original Columbia Card Task, where the number of loss cards typically produces the strongest and most consistent effects on decision-making compared to other task parameters (Figner et al., 2009). This suggests that the likelihood rather than the severity of potential harm to others drives differential responses to social exclusion, indicating that antisocial decision-making may be particularly sensitive to probability-based rather than magnitude-based risk information.

That is, well-accepted adolescents becoming more cautious and poorly accepted adolescents becoming more risk-taking when potential costs to others increase—while those with moderate classroom social acceptance showed less sensitivity to risk information when in an acute situation of social exclusion. This prioritization of self-relevant over other-relevant information depending on the broad social context aligns with dual-process models of decision-making (Kahneman and Frederick, 2002), suggesting that social exclusion may differentially affect the systems related to personal reward detection and deliberative risk assessment for others.

Rather than supporting the general cognitive depletion hypothesis (Baumeister et al., 2002, 2005; Walasek et al., 2019), our findings suggest strategic rather than impaired cognitive processing. Poorly integrated adolescents demonstrate deliberate prioritization of self-beneficial information while selectively disregarding information about potential harm to others when excluded. This pattern indicates purposeful decision-making rather than general cognitive impairment.

The antisocial risk-taking patterns we observed connect with research characterizing bullying as strategic, goal-directed behavior rather than simply impulsive aggression (Olthof et al., 2011; Peeters et al., 2010). The specific pattern displayed by poorly integrated adolescents—the willingness to accept higher risks of harming others for personal gain when excluded—corresponds with the conceptualization of antisocial risk-taking as behavior that maximizes personal benefits while disregarding potential harm to others (Do et al., 2017). This parallels findings that adolescents with bullying tendencies often demonstrate selective rather than general deficits in decision-making, specifically showing problems with considering others’ welfare rather than engaging in indiscriminate risk-taking (Flouri and Papachristou, 2019).

Our exploratory analysis of moral disengagement provides valuable context for understanding antisocial behavior patterns. Moral disengagement has been consistently linked with various forms of antisocial conduct, including aggression and bullying (Killer et al., 2019; Mazzone et al., 2021; Obermann, 2013). Our finding that moral disengagement predicted antisocial risk-taking suggests these cognitive mechanisms may enable antisocial tendencies by neutralizing concerns about harming others that would typically constrain such behaviors.

The relationship between moral disengagement and social integration reveals a compelling pattern. We found a significant negative correlation between these variables. This suggests that adolescents with poor social integration are more likely to employ moral disengagement strategies, potentially creating a pathway where social marginalization facilitates antisocial behavior through reduced moral constraints.

Interestingly, our data revealed an unexpected pattern regarding experimental conditions: participants in the inclusion condition reported slightly higher levels of moral disengagement compared to those in the exclusion condition, though this difference was only marginally significant. This counterintuitive finding warrants careful interpretation. Several possibilities may explain this pattern: First, the measurement sequence (moral disengagement assessed after both the experimental manipulation and risk-taking task) may have influenced these results. Included participants who engaged in antisocial risk-taking might have subsequently activated moral disengagement mechanisms to reconcile their prosocial inclusion experience with their antisocial choices. Excluded participants, conversely, might have felt less need to justify harmful actions, as their behavior could already be framed as a response to perceived mistreatment. Second, inclusion experiences might create a sense of social security that paradoxically enables greater moral disengagement through mechanisms like diffusion of responsibility (Gini et al., 2014). Included adolescents might find it easier to distribute accountability for antisocial actions across their perceived group, reducing individual feelings of responsibility. Meanwhile, excluded participants might experience heightened self-awareness, making it more difficult to displace personal responsibility.

Rather than undermining our findings, this unexpected pattern conceptually works against our hypotheses yet still supports our main conclusion: despite these marginal group differences, the relationship between higher moral disengagement and increased antisocial risk-taking remains robust. This suggests moral disengagement functions as a relatively enduring cognitive framework that mediates responses to social situations rather than a malleable state that fluctuates with immediate social experiences. This interpretation aligns with developmental perspectives suggesting that patterns like moral disengagement develop gradually through repeated social interactions (Bandura, 1999; Perren et al., 2012) rather than arising spontaneously in response to isolated social events.

### 4.3 Towards an integrated model of social exclusion and antisocial behavior

Our findings suggest an integrated model where both social reconnection theory and frustration-aggression hypothesis operate simultaneously but manifest differently based on social acceptance status. Well-accepted adolescents show adaptive responses consistent with social reconnection theory, while poorly integrated adolescents demonstrate defensive responses supporting the frustration-aggression hypothesis

Regarding chronic social exclusion, our findings align with developmental models that emphasize the cumulative effects of persistent social marginalization (Arseneault et al., 2010; Stenseng et al., 2015). The significant correlation between low classroom social acceptance and moral disengagement, supports the social cognitive theory (Bandura, 1999) which suggests that chronic negative social experiences may gradually foster cognitive mechanisms that facilitate antisocial behavior. This relationship between relatively lower classroom social acceptance and moral disengagement echoes findings that identified similar connections between social marginalization and moral justification processes (Mazzone et al., 2021).

Our results also suggest a theoretical model in which responses to social exclusion follow a curvilinear rather than linear relationship with classroom social acceptance. Both well-accepted and poorly accepted adolescents showed enhanced sensitivity to exclusion but in opposite directions, while those with moderate classroom acceptance showed less pronounced responses. This U-shaped pattern may reflect different underlying psychological processes: For well-accepted adolescents, exclusion may represent an unexpected threat to a valued social position, triggering protective responses. In contrast, for poorly accepted adolescents, exclusion may confirm chronically negative social experiences, amplifying existing defensive tendencies. This theoretical perspective helps reconcile conflicting findings in previous research by suggesting that sample characteristics regarding social acceptance may determine which behavioral tendencies predominate.

From a practical perspective, our findings offer an encouraging outlook for educational interventions. While adolescents will inevitably experience some degree of social exclusion in peer contexts, our results suggest that fostering strong classroom social integration may serve as a protective factor that promotes adaptive rather than antisocial responses to such experiences. This highlights the critical importance of creating inclusive school environments that support all students’ social integration as a preventive approach to antisocial behavior, rather than focusing solely on managing exclusion experiences themselves. These findings are particularly relevant during adolescence, when social status concerns are heightened and peer relationships profoundly shape developmental trajectories (Blakemore, 2018; Eccles and Roeser, 2011).

This integrated model has several advantages over single-process theories. First, it accounts for the heterogeneity in responses to social exclusion observed across different studies by explicitly incorporating classroom social acceptance as a key moderating factor. Second, it bridges social-psychological theories of immediate exclusion effects with developmental perspectives on chronic marginalization, providing a more comprehensive framework for understanding how both acute and chronic exclusion experiences shape antisocial tendencies. Third, it connects information processing approaches with moral cognition research, suggesting that antisocial behavior involves both selective attention to different types of information and moral disengagement processes that justify potential harm to others.

### 4.4 Strengths, limitations, and future directions

Our study offers several methodological contributions to research on social exclusion and antisocial behavior. By combining sociometric assessment of classroom social acceptance with experimental manipulation of acute social exclusion, we disentangled the effects of long-term social standing from immediate exclusion experiences. The antisocial risk-taking paradigm we developed provides a more precise measure of the calculated aspects of behavior that often characterize bullying interactions (Olthof et al., 2011; Peeters et al., 2010).

Despite these methodological strengths, several limitations warrant consideration. First, our sample was drawn from a specific geographic region in Germany with adolescents from Years 12–16, potentially limiting generalizability to other cultural contexts. It is also important to note that our measure of classroom social acceptance captured natural variation in classroom social standing rather than severe cases of chronic social exclusion. This distinction highlights that even relatively moderate differences in classroom social acceptance—not necessarily reaching the level of clinical or severe social isolation—were sufficient to influence socio-cognitive processing in antisocial risk-taking. This suggests that the effects of classroom social acceptance on antisocial tendencies may operate along a continuum, with even subtle differences in social standing potentially shaping how adolescents process and respond to social information.

Additionally, while our experimental design established relationships between acute social exclusion and subsequent behavior, the single-session nature prevents examination of how these patterns might evolve over time. Our antisocial risk-taking paradigm may not fully capture the complex social considerations that influence antisocial behavior in naturalistic settings and does not allow for distinguishing between different behavioral motivations. Participants who selected fewer cards might have done so due to genuine concern for others, adherence to social norms, strategic self-presentation, or risk aversion for different reasons.

A critical limitation also concerns the temporal sequence of our measurements. Because we assessed moral disengagement after both the social exclusion manipulation and the antisocial risk-taking task, we cannot determine the direction of the relationship between moral disengagement and antisocial behavior. While we interpret moral disengagement as a relatively stable cognitive tendency that influences antisocial risk-taking, it is equally possible that participants’ experiences during the social exclusion manipulation and their subsequent choices in the risk-taking task influenced how they responded to the moral disengagement questions. For example, participants who engaged in more antisocial risk-taking may have subsequently activated moral disengagement mechanisms to justify their behavior, or the exclusion experience itself may have temporarily increased their willingness to morally disengage. This measurement sequence prevents us from establishing whether moral disengagement predisposes individuals to antisocial behavior or whether antisocial behavior and social exclusion experiences lead to increased moral disengagement as a post-hoc justification mechanism.

It is also important to note that our cross-sectional design does not allow us to definitively establish the direction between classroom social acceptance and antisocial responding. While we interpret classroom social acceptance as a potential protective factor, an equally plausible interpretation is that underlying social competency deficits contribute to both poor classroom acceptance and maladaptive responses to exclusion. This alternative explanation aligns with social skills deficit models, suggesting that adolescents with poor social competencies may be both more likely to experience chronic peer rejection and more prone to antisocial responses when faced with additional social threats.

Additionally, our focus on classroom social acceptance may not capture other important aspects of adolescents’ social experiences, such as friendships outside the classroom or online social connections. Finally, due to time constraints during data collection, we did not include a manipulation check such as mood ratings or explicit questions about feelings of inclusion/exclusion following the Cyberball paradigm. However, the robust effectiveness of this established manipulation in previous research (Hartgerink et al., 2015) and our own prior work (Lorenz and Ferdinand, 2025), combined with the significant interaction effects involving our exclusion condition, suggest that the experimental manipulation successfully influenced participants’ responses in theoretically meaningful ways.

Future research should address these limitations through longitudinal designs that track how social acceptance, exclusion experiences, and antisocial tendencies develop over time. Cross-cultural studies and examinations across different developmental periods would enhance understanding of how cultural norms and developmental stages influence these relationships. The connection between moral disengagement and antisocial risk-taking warrants further investigation, particularly regarding potential interventions. Expanding research to include a spectrum of risk-taking paradigms—prosocial (benefiting others), self-oriented (personal outcomes only), and antisocial—would provide a more comprehensive understanding of how social exclusion influences decision-making across contexts. Complementing experimental approaches with naturalistic observations would enhance ecological validity. Finally, exploring additional moderators and mediators, such as emotion regulation abilities, executive functioning, or family support, could further refine our understanding of how social experiences influence antisocial tendencies in adolescence.

## 5 Conclusion

This study advances our understanding of the complex relationship between social exclusion, classroom social acceptance, and antisocial behavior in adolescents. Our findings demonstrate that responses to social exclusion are not uniform but depend critically on pre-existing classroom social acceptance status, with well-accepted and poorly accepted adolescents showing distinctly different patterns of risk sensitivity and decision-making following exclusion experiences. The significant three-way interaction between classroom social acceptance, social exclusion, and risk parameters reveals a complex pattern where social factors fundamentally alter how adolescents process information in antisocial contexts.

Our results challenge simplistic views of antisocial behavior by revealing specific information processing patterns—particularly how adolescents with different levels of classroom social acceptance selectively attend to self-relevant versus other-relevant information following exclusion. This differential sensitivity to risk information, combined with our exploratory findings on moral disengagement, highlights potential cognitive mechanisms that may play a key role in the development of antisocial tendencies. This mechanistic understanding provides an important foundation for future research examining the developmental trajectories of these cognitive processes and their relationship to social experiences, potentially identifying critical points where maladaptive processing patterns might be addressed before they consolidate into stable behavioral tendencies.

## Data Availability

The raw data supporting the conclusions of this article will be made available by the authors, without undue reservation.

## Footnotes

^1^The 12 participants were excluded from analysis because they showed extremely limited response variability (< 2 different card values across all trials), indicating that they did not engage with the task as intended. Specifically, 10 of these participants consistently selected only the minimum number of cards (1 card) required to proceed through each trial, while two others showed similarly restricted response patterns (one person always choosing one or two cards and another always choosing all 32 cards). This pattern suggests that these participants did not take the task seriously and did not pay attention to the instructions.

## References

[ref1] Arseneault L. Bowes L. Shakoor S. (2010). Bullying victimization in youths and mental health problems: ‘much ado about nothing’? Psychol. Med. 40, 717–729. doi: 10.1017/S0033291709991383, PMID: 19785920

[ref2] Averdijk M. Malti T. Eisner M. Ribeaud D. Farrington D. P. (2016). A vicious cycle of peer victimization? Problem behavior mediates stability in peer victimization over time. J. Dev. Life-Course Criminol. 2, 162–181. doi: 10.1007/s40865-016-0024-7

[ref3] Bandura A. (1999). Moral disengagement in the perpetration of inhumanities. Personal. Soc. Psychol. Rev. 3, 193–209. doi: 10.1207/s15327957pspr0303_3, PMID: 15661671

[ref9001] Barr D. J. Levy R. Scheepers C. Tily H. J. (2013). Random effects structure for confirmatory hypothesis testing: Keep it maximal. Journal of Memory and Language, 68, 255–278. doi: 10.1016/j.jml.2012.11.001 PMC3881361 24403724

[ref9002] Bates D. Mächler M. Bolker B. Walker S. (2015). Fitting Linear Mixed-Effects Models Using lme4. Journal of Statistical Software, 67. doi: 10.18637/jss.v067.i01

[ref4] Baumeister R. F. DeWall C. N. Ciarocco N. J. Twenge J. M. (2005). Social exclusion impairs self-regulation. J. Pers. Soc. Psychol. 88, 589–604. doi: 10.1037/0022-3514.88.4.589, PMID: 15796662

[ref5] Baumeister R. F. Twenge J. M. Nuss C. K. (2002). Effects of social exclusion on cognitive processes: anticipated aloneness reduces intelligent thought. J. Pers. Soc. Psychol. 83, 817–827. doi: 10.1037/0022-3514.83.4.817, PMID: 12374437

[ref6] Blakemore S.-J. (2018). Avoiding social risk in adolescence. Curr. Dir. Psychol. Sci. 27, 116–122. doi: 10.1177/0963721417738144

[ref7] Blakemore S.-J. Mills K. L. (2014). Is adolescence a sensitive period for sociocultural processing? Annu. Rev. Psychol. 65, 187–207. doi: 10.1146/annurev-psych-010213-115202, PMID: 24016274

[ref8] Calkins S. D. Keane S. P. (2009). Developmental origins of early antisocial behavior. Dev. Psychopathol. 21, 1095–1109. doi: 10.1017/S095457940999006X, PMID: 19825259 PMC2782636

[ref9] Casey B. J. (2015). Beyond simple models of self-control to circuit-based accounts of adolescent behavior. Annu. Rev. Psychol. 66, 295–319. doi: 10.1146/annurev-psych-010814-015156, PMID: 25089362

[ref10] Cillessen A. H. N. (2009). “Sociometric methods” in Handbook of peer interactions, relationships, and groups. eds. Rubin K. H. Bukowski W. M. Laursen B. (The Guilford Press. Guilford), 82–99.

[ref11] Crick N. R. Dodge K. (1994). A review and reformulation of social information-processing mechanisms in children’s social adjustment. Psychol. Bull. 115, 74–101. doi: 10.1037/0033-2909.115.1.74

[ref13] Do K. T. Guassi Moreira J. F. Telzer E. H. (2017). But is helping you worth the risk? Defining prosocial risk taking in adolescence. Dev. Cogn. Neurosci. 25, 260–271. doi: 10.1016/j.dcn.2016.11.008, PMID: 28063823 PMC5461219

[ref14] Eccles J. S. Roeser R. W. (2011). “School and community influences on human development” in Developmental science: An advanced textbook. eds. Bornstein M. H. Lamb E.. 6th ed. (New York: Psychology Press), 571–643.

[ref15] Faul F. Erdfelder E. Buchner A. Lang A.-G. (2009). Statistical power analyses using G*power 3.1: tests for correlation and regression analyses. Behav. Res. Methods 41, 1149–1160. doi: 10.3758/BRM.41.4.1149, PMID: 19897823

[ref16] Figner B. Mackinlay R. J. Wilkening F. Weber E. U. (2009). Affective and deliberative processes in risky choice: age differences in risk taking in the Columbia card task. J. Exp. Psychol. Learn. Mem. Cogn. 35, 709–730. doi: 10.1037/a0014983, PMID: 19379045

[ref17] Flouri E. Papachristou E. (2019). Peer problems, bullying involvement, and affective decision-making in adolescence. Br. J. Dev. Psychol. 37, 466–485. doi: 10.1111/bjdp.12287, PMID: 30973653

[ref18] Frick P. J. Viding E. (2009). Antisocial behavior from a developmental psychopathology perspective. Dev. Psychopathol. 21, 1111–1131. doi: 10.1017/S0954579409990071, PMID: 19825260

[ref19] Gini G. Pozzoli T. Hymel S. (2014). Moral disengagement among children and youth: a meta-analytic review of links to aggressive behavior. Aggress. Behav. 40, 56–68. doi: 10.1002/ab.21502, PMID: 24037754

[ref9004] Hartgerink C. H. J. van Beest I. Wicherts J. M. Williams K. D. (2015). The Ordinal Effects of Ostracism: A Meta-Analysis of 120 Cyberball Studies. PLOS ONE, 10, e0127002. doi: 10.1371/journal.pone.0127002 26023925 PMC4449005

[ref20] Kahneman D. Frederick S. (2002). “Representativeness revisited: attribute substitution in intuitive judgment” in Heuristics and biases (Cambridge: Cambridge University Press), 49–81. doi: 10.1017/CBO9780511808098.004

[ref21] Killer B. Bussey K. Hawes D. J. Hunt C. (2019). A meta-analysis of the relationship between moral disengagement and bullying roles in youth. Aggress. Behav. 45, 450–462. doi: 10.1002/ab.21833, PMID: 30900277

[ref22] Kuznetsova A. Brockhoff P. B. Christensen R. H. B. (2017). lmerTest package: tests in linear mixed effects models. J. Stat. Softw. 82:13. doi: 10.18637/jss.v082.i13

[ref23] Leary M. R. Kowalski R. M. Smith L. Phillips S. (2003). Teasing, rejection, and violence: case studies of the school shootings. Aggress. Behav. 29, 202–214. doi: 10.1002/ab.10061

[ref24] Lenth R. V. (2025). Emmeans: estimated marginal means, aka least-squares means (R package version 1.11.1-00001). Available online at: https://rvlenth.github.io/emmeans/ (accessed July 14, 2025).

[ref25] Lorenz C. Ferdinand N. K. (2025). Combined effects of social exclusion and social rank feedback on risky decision-making across adolescence. J. Youth Adolesc. 54, 537–558. doi: 10.1007/s10964-024-02072-w, PMID: 39198345 PMC11846755

[ref26] MacDonald G. Leary M. R. (2005). Why does social exclusion hurt? The relationship between social and physical pain. Psychol. Bull. 131, 202–223. doi: 10.1037/0033-2909.131.2.202 15740417

[ref27] Maner J. K. DeWall C. N. Baumeister R. F. Schaller M. (2007). Does social exclusion motivate interpersonal reconnection? Resolving the "porcupine problem.". J. Pers. Soc. Psychol. 92, 42–55. doi: 10.1037/0022-3514.92.1.42, PMID: 17201541

[ref28] Mazzone A. Yanagida T. Camodeca M. Strohmeier D. (2021). Information processing of social exclusion: links with bullying, moral disengagement and guilt. J. Appl. Dev. Psychol. 75:101292. doi: 10.1016/j.appdev.2021.101292, PMID: 40748484

[ref29] Mazzone A. Yanagida T. Caravita C. S. Strohmeier D. (2019). Moral emotions and moral disengagement: concurrent and longitudinal associations with aggressive behavior among early adolescents. J. Early Adolesc. 39, 839–863. doi: 10.1177/0272431618791276

[ref30] Medeiros W. Torro-Alves N. Malloy-Diniz L. F. Minervino C. M. (2016). Executive functions in children who experience bullying situations. Front. Psychol. 7, 1–9. doi: 10.3389/fpsyg.2016.01197, PMID: 27616998 PMC5000580

[ref31] Moreno J. L. (1953). Who shall survive? Foundations of sociometry, group psychotherapy and sociodrama. Beacon, NY: Beacon House Inc.

[ref32] Newman M. L. (2014). Here we go again: bullying history and cardiovascular responses to social exclusion. Physiol. Behav. 133, 76–80. doi: 10.1016/j.physbeh.2014.05.014, PMID: 24858188

[ref33] Obermann M.-L. (2013). Temporal aspects of moral disengagement in school bullying: crystallization or escalation? J. Sch. Violence 12, 193–210. doi: 10.1080/15388220.2013.766133

[ref34] Olthof T. Goossens F. A. Vermande M. M. Aleva E. A. Meulen M. V. D. (2011). Bullying as strategic behavior: relations with desired and acquired dominance in the peer group. J. Sch. Psychol. 49, 339–359. doi: 10.1016/j.jsp.2011.03.003, PMID: 21640248

[ref35] Olweus D. (1993). Bullying at school: what we know and what we can do. Wiley-Blackwell.

[ref36] Peeters M. Cillessen A. H. N. Scholte R. H. J. (2010). Clueless or powerful? Identifying subtypes of bullies in adolescence. J. Youth Adolesc. 39, 1041–1052. doi: 10.1007/s10964-009-9478-9, PMID: 20625880

[ref37] Pepler D. Jiang D. Craig W. Connolly J. (2008). Developmental trajectories of bullying and associated factors. Child Dev. 79, 325–338. doi: 10.1111/j.1467-8624.2007.01128.x, PMID: 18366426

[ref38] Perren S. Gutzwiller-Helfenfinger E. Malti T. Hymel S. (2012). Moral reasoning and emotion attributions of adolescent bullies, victims, and bully-victims. Br. J. Dev. Psychol. 30, 511–530. doi: 10.1111/j.2044-835X.2011.02059.x, PMID: 23039330

[ref39] R Core Team (2023). R: A language and environment for statistical computing. Vienna: R Foundation for Statistical Computing.

[ref40] Reijntjes A. Thomaes S. Bushman B. J. Boelen P. A. Castro B. O. D. Telch M. J. (2010). The outcast-lash-out effect in youth: alienation increases aggression following peer rejection. Psychol. Sci. 21, 1394–1398. doi: 10.1177/0956797610381509, PMID: 20739674

[ref41] Reijntjes A. Vermande M. Olthof T. Goossens F. A. Van de Schoot R. Aleva L.. (2013). Costs and benefits of bullying in the context of the peer group: a three wave longitudinal analysis. J. Abnorm. Child Psychol. 41, 1217–1229. doi: 10.1007/s10802-013-9759-3, PMID: 23686239

[ref42] Ribeaud D. Eisner M. (2010). Are moral disengagement, neutralization techniques, and self-serving cognitive distortions the same? Developing a unified scale of moral neutralization of aggression. Int. J. Confl. Violence 4, 298–315.

[ref43] Riva P. Eck J. (2016) in Social exclusion: Psychological approaches to understanding and reducing its impact. eds. Riva P. Eck J. (Cham: Springer International Publishing).

[ref45] Smart Richman L. Leary M. R. (2009). Reactions to discrimination, stigmatization, ostracism, and other forms of interpersonal rejection: a multimotive model. Psychol. Rev. 116, 365–383. doi: 10.1037/a0015250, PMID: 19348546 PMC2763620

[ref46] Somerville L. H. (2013). The teenage brain: sensitivity to social evaluation. Curr. Dir. Psychol. Sci. 22, 121–127. doi: 10.1177/0963721413476512, PMID: 24761055 PMC3992953

[ref47] Stenseng F. Belsky J. Skalicka V. Wichstrøm L. (2014). Preschool social exclusion, aggression, and cooperation: a longitudinal evaluation of the need-to-belong and the social-reconnection hypotheses. Personal. Soc. Psychol. Bull. 40, 1637–1647. doi: 10.1177/0146167214554591, PMID: 25304257

[ref48] Stenseng F. Belsky J. Skalicka V. Wichstrøm L. (2015). Social exclusion predicts impaired self-regulation: a 2-year longitudinal panel study including the transition from preschool to school. J. Pers. 83, 212–220. doi: 10.1111/jopy.12096, PMID: 24635533

[ref49] Timeo S. Riva P. Paladino M. P. (2019). Learning to cope with everyday instances of social exclusion: a review of emotional and cognitive strategies for children and adolescents. J. Appl. Biobehav. Res. 24, 1–17. doi: 10.1111/jabr.12173, PMID: 40740696

[ref50] Tomova L. Andrews J. L. Blakemore S.-J. (2021). The importance of belonging and the avoidance of social risk taking in adolescence. Dev. Rev. 61:100981. doi: 10.1016/j.dr.2021.100981

[ref51] Twenge J. M. Baumeister R. F. DeWall C. N. Ciarocco N. J. Bartels J. M. (2007). Social exclusion decreases prosocial behavior. J. Pers. Soc. Psychol. 92, 56–66. doi: 10.1037/0022-3514.92.1.56, PMID: 17201542

[ref52] Twenge J. M. Baumeister R. F. Tice D. M. Stucke T. S. (2001). If you can’t join them, beat them: effects of social exclusion on aggressive behavior. J. Pers. Soc. Psychol. 81, 1058–1069. doi: 10.1037/0022-3514.81.6.1058, PMID: 11761307

[ref54] Walasek L. Juanchich M. Sirota M. (2019). Adaptive cooperation in the face of social exclusion. J. Exp. Soc. Psychol. 82, 35–46. doi: 10.1016/j.jesp.2018.11.005, PMID: 40748484

[ref55] Warburton W. A. Williams K. D. Cairns D. R. (2006). When ostracism leads to aggression: the moderating effects of control deprivation. J. Exp. Soc. Psychol. 42, 213–220. doi: 10.1016/j.jesp.2005.03.005

[ref57] Williams K. D. Jarvis B. (2006). Cyberball: a program for use in research on interpersonal ostracism and acceptance. J. Behav. Res. Methods 38, 174–180. doi: 10.3758/BF03192765, PMID: 16817529

